# *QuickStats:* Percentage* of Adults Aged ≥18 Years Who Had Visited an Emergency Department at Least Once in the Past 12 Months,^†^ by Age Group and Inflammatory Bowel Disease (IBD) Status^§^ — National Health Interview Survey, 2015 and 2016^¶^

**DOI:** 10.15585/mmwr.mm6808a6

**Published:** 2019-03-01

**Figure:**
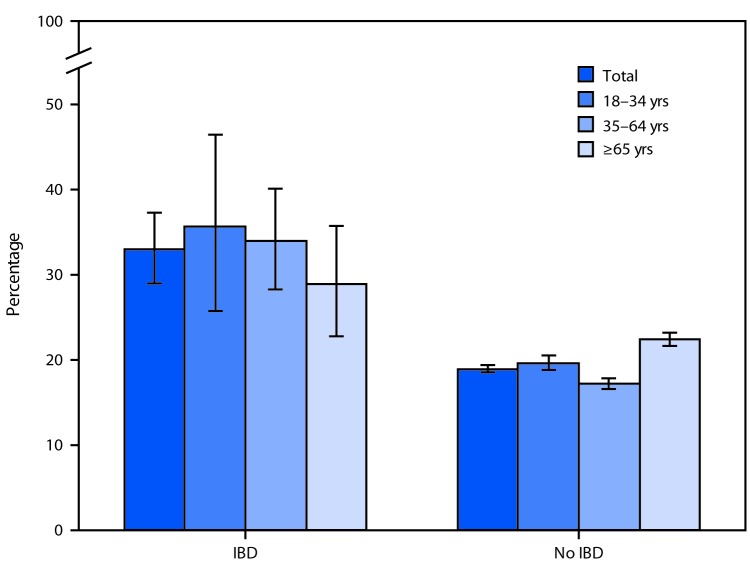
In 2015 and 2016, adults with IBD were more likely to have visited an emergency department at least once in the past 12 months than were those without IBD (33.0% versus 18.9%); this pattern was observed for all age groups. Among adults aged 18–34, 35–64, and ≥65 years, those with IBD were more likely to have visited an emergency department at least once in the past 12 months (35.6%, 34.0%, and 28.9%, respectively), compared with adults without IBD (19.6%, 17.2%, and 22.4%, respectively).

**Source:** National Health Interview Survey, 2015 and 2016 data. https://www.cdc.gov/nchs/nhis.htm.

